# Systemic Lupus Erythematosus-Related Pancreatitis in Children: Severe and Lethal Form

**DOI:** 10.1155/2018/4612754

**Published:** 2018-12-31

**Authors:** R. El Qadiry, A. Bourrahouat, I. Aitsab, M. Sbihi, Y. Mouaffak, F. Z. Moussair, S. Younous

**Affiliations:** ^1^Pediatric B Department, Mother-Child Pole, Mohammed VI University Hospital, Marrakesh, Morocco; ^2^Pediatric ICU Department, Mother-Child Pole, Mohammed VI University Hospital, Marrakesh, Morocco

## Abstract

Systemic lupus erythematosus (SLE) is a chronic autoimmune inflammatory disease of unknown cause, characterized by multisystemic involvement. Its occurrence in children is rare, and acute pancreatitis is exceptional in this matter. Its diagnosis is clinical, biological, and radiological. Its treatment is based on corticosteroid therapy, and its progress is generally lethal. We report two cases of acute pancreatitis in the course of SLE, highlighting its life-threatening severity despite well-conducted treatment. *Case 1*: 14-year-old patient, admitted to the pediatric ICU for altered state of consciousness. This child, an outpatient since 2009 for chronic arthralgia, was hospitalized five days previously in the pediatric ward for suspicion of severe SLE, before presenting abdominal pain and vomiting. Hyperlipasemia was found, and an abdominal CT scan confirmed the diagnosis of acute pancreatitis. The patient was put under immunosuppressive therapy composed of high-dosage of corticosteroid and cyclophosphamide cures. She died 20 days after her hospitalization by severe lupus flare with multiorgan failure. *Case 2*: 14-year-old child, admitted to the Pediatric ward for prolonged fever associated with polyarthralgia (nondeforming, immovable, and additive) that had been progressing since 6 months with altered general state; his symptoms got worst 15 days before his hospitalization by having behavioral disorders and epigastralgia with vomiting. Pancreatitis was strongly suspected in the absence of improvement on symptomatic treatment and confirmed by hyperlipasemia 6 times the normal value and a swollen pancreas on the abdominal CT scan. The child was treated with Solumedrol and cyclophosphamide without improvement and then died after one month of hospitalization by a septic shock.

## 1. Introduction

Systemic lupus erythematosus (SLE) is a chronic autoimmune inflammatory disease, characterized by the existence of an array of autoantibodies, as well as the creation of immune complexes. It can be as early as childhood in 15 to 20% of cases, or even from the age of three years. Although gastrointestinal symptoms are frequent, acute pancreatitis remains exceptional, since less than 100 cases have been reported in the literature [1]. Apart from its rarity and life-threatening severity despite well-conducted treatment, it is not usual for this condition to be revealing of SLE [2]. Authors present two clinical observations of SLE in two young teenagers, revealed by acute pancreatitis, and review its epidemiological, clinical, physiopathological, and therapeutic characteristics, as well as its prognosis.

## 2. Case 1

A 14-year-old girl was hospitalized in pediatric ICU for abdominal pain, respiratory distress, and progressive alteration of her consciousness. This patient with no medical history or family history of autoimmune disease has been suffering from chronic arthralgia for five years. She was first admitted to the pediatric ward before being transferred five days later to pediatric ICU for clinical exacerbation.

Upon admission, she opened her eyes spontaneously, executed orders but had illogical speech. She was normotensive, tachycardiac at 105 bpm, and tachypneic at 35 cpm, and her peripheral oxygen saturation was 92% at room air. Cardiothoracic auscultation had revealed muted heart sounds and bilateral pleural effusion syndrome. The abdominal examination had found epigastric tenderness and ascites of moderate quantity. In addition, generalized edematous syndromes associated with cutaneous signs were noted (malar erythema, alopecia, and pulpitis of the fingers and toes).

The biological assessment showed a normochromic normocytic anemia at 9.9 g/dl, leukocytosis at 16900 elements/mm^3^, and thrombocytopenia at 62000/mm^3^, as well as an impairment of renal function, with urea at 1, 06 g/l and creatinine at 61 mg/l. Her natremia was collapsed (106 mmol/l), and her calcemia and albumin too.

**Figure 1:**
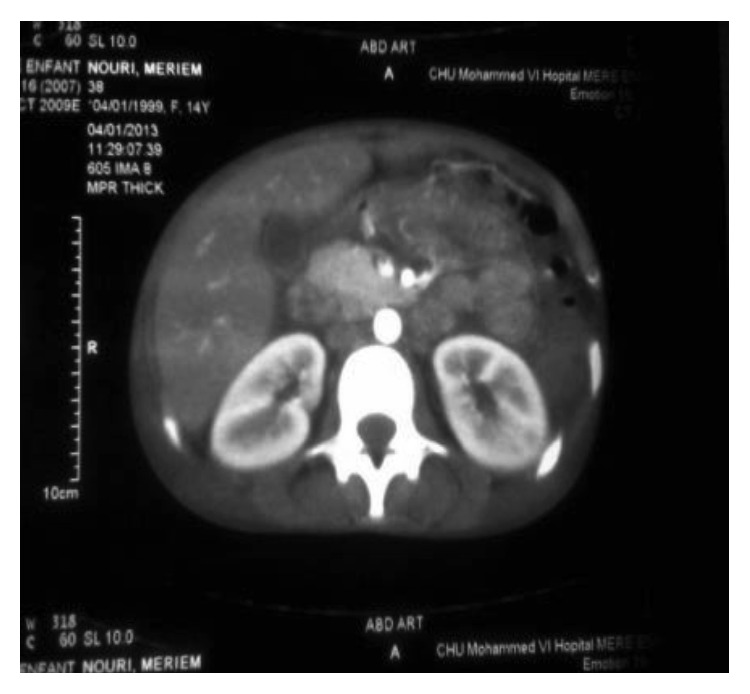
PA stage C of Balthazar.

**Figure 2:**
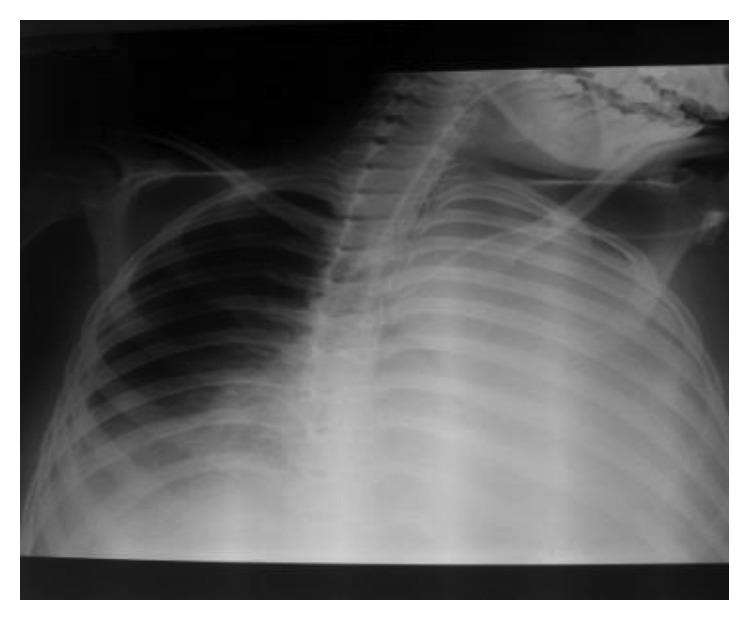
Pleural effusion.

**Figure 3:**
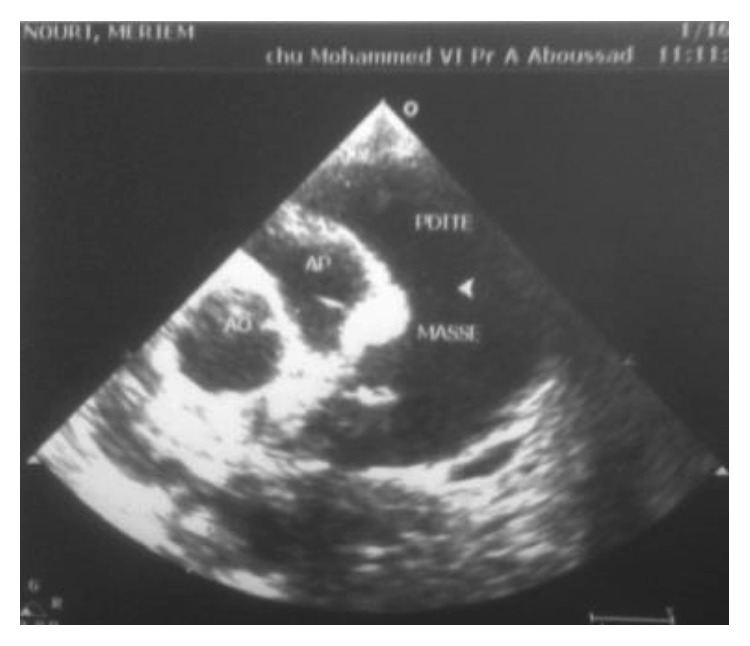
Pericarditis.

The diagnosis of pancreatitis was based on the measurement of lipasemia at 810 IU/L, with an edematous pancreas on abdominal ultrasonography and intrapancreatic necrosis on abdominal CT (stage C of Balthazar) (Figure 1). Radiological and ultrasonographic investigations confirmed the presence of pleural and pericardial effusion (Figures 2 and 3).

In front of the multisystemic symptoms and following the criteria of the American College of Rheumatology (ACR), an immunological assessment was carried out and the diagnosis of SLE was retained with the existence of nonerosive arthritis, pleurisy and pericarditis, neurological involvement (seizures) renal involvement, hematologic abnormalities as well as immunological disorders confirmed by the decreased complement factors C3 and C4, and the positivity of anti-RNP antibodies, anti-Sm antibodies, anti-SSA as well as anti-SSB; however, antiphospholipid antibodies were negative.

The plan of management started by the correction of metabolic disorders and renal function, and the initiation of an immunosuppressive therapy was based on steroids and cyclophosphamides as a bolus. She died 20 days after her hospitalization by a severe lupus flare with multiorgan failure.

## 3. Case 2

A 14-year-old girl, with no particular history, admitted to the Pediatric ward for prolonged fever associated with polyarthralgia (nondeforming, immovable, and additive) that had been progressing since 6 months with altered general state. His symptoms got worst 15 days before his hospitalization by having headache, behavioral disorders like agitation, and severe epigastralgia with vomiting.

On admission: clinical examination found a confused patient, feverish at 38.5°C, normotensive, tachycardic at 125 bpm, and tachypneic at 36 cpm. Also noted the existence of skin rash on the face, mouth ulcers bleeding on contact, pain in both passive and active mobility in large joints, no inflammatory signs, and general abdominal tenderness. The rest of the somatic examination including the neurological examination was ordinary. Biologically, normochromic normocytic anemia was observed at 7.2 g/dl without signs of haemolysis, thrombocytopenia at 86 000/*μ*l and lymphopenia at 1200/*μ*l, SV at 50 mm at the first hour, and CRP at 69 mg/l, and proteinuria 24 to 16 mg/kg/24 h and normal renal function.

The diagnosis of pancreatitis was strongly suspected and confirmed by hyperlipasemia at 610 IU/L and a swollen form of the pancreas on abdominal CT scan. Cerebral MRI was also mandatory in front of persistent headache and found signal abnormality of subtentorial white matter of the left frontoparietal and right occipital that could be part of neurolupus.

The diagnosis of SLE was retained in front of the multisystemic symptoms and meeting the criteria of the American College of Rheumatology (ACR). The antinuclear antibodies, anti-Sm, and native anti-DNAs were positive associated with C3 hypocomplementemia.

The child was treated with bolus of Solumedrol and cyclophosphamide beside his symptomatic treatment without improvement and then died after one month of hospitalization by a septic shock.

## 4. Discussion

These two cases illuminate a rare manifestation of SLE which is acute pancreatitis (AP). The aim of our work is on one hand to show that SLE can be extremely rarely exposed by acute AP, on the other hand, is how this clinical presentation revealed in the child.

AP is an extremely rare and potentially lethal manifestation of SLE. The incidence of AP associated with SLE varies from 0.7 to 4% [2–5]. The link between lupus and pancreatitis was first documented in 1939 by Reifenstein and al [2, 3]. Since, only about 99 cases of pancreatitis on SLE have been described in the literature, 10 patients presented pancreatitis as initial expression [3, 4, 6].

The pathogenic mechanism of AP in SLE is complex and multifactorial; it includes vasculitis, interstitial edema, and immune complex deposition associated with arterial or arteriolar occlusions. Other physiopathological hypotheses have been put forward, mainly the production of autoantibodies, an abnormal immune response of pancreatic cells, and drug toxicity [3, 6].

The diagnosis of pancreatitis on SLE can only be made after elimination of other causes of acute pancreatitis. Viral causes of HIV are included in immunocompromised patients [3, 4].

Both of our patients had multiple-organ involvement in their lupus, such as hematologic, renal, neurological, gastrointestinal, and dermatological, but the most distinctive feature of their clinical pictures was the pancreatitis.

The direct diagnosis of pancreatitis is usually based on clinical signs of abdominal pain, nausea, and vomiting, supported by lab results of abnormal pancreatic enzymes and tomographic signs in favor. These symptoms can also be described for other gastrointestinal disease or side effects of multiple therapies, which can lead to misguidance. It has been reported that these clinical signs were wrongly accredited to etiologies other than AP in 88.6% [3, 7]. This delayed diagnosis and inadequate treatment contributed to the already known critical and potentially lethal prognosis of the disease [3, 8].

Clinically, AP symptoms can range from simple benign form with self-limitation of the disease to severe form with fulminant progression. In the SLE, in addition to the obvious cases of AP, there were also cases of subclinical pancreatitis with elevation of pancreatic enzymes without clinical symptoms. The incidence of subclinical pancreatitis may be significantly higher than that of clinical pancreatitis. One study found hyperamylasemia in 30.5% of SLE in asymptomatic patients. The diagnosis is made by CT in 70% to 90% of cases [3, 9].

Both patients had abdominal pain, nausea, vomiting, and remarkably high levels of pancreatic enzymes, and abdominal CT findings concurred with the diagnosis of AP.

Gormezano et al. reported high mortality in children with SLE and AP. Indeed, in their large cohort, among 13 patients with SLE and AP, with an average age of 13.5 years, 4 (31%) died, whereas none of the adult patients with SLE and AP had had a lethal problem [10]. Yang et al. also described mortality rates that are significantly linked to SLE-acute pancreatitis association [3]. These rates are more important when in addition we have thrombocytopenia, leukopenia, hypoalbuminemia, or hematuria [3]. The mortality rate of AP in pediatric cases of SLE was higher when several organs are involved, in particular renal, hepatic, and neurological. Richer et al. reported that 57% of pediatric SLE had severe cases of AP with 45% of death rate [3, 11].

Articular and neurological involvement is significantly more frequent in patients with AP during SLE, and this could be explained by lupus disease with relatively severe flare [12]. The absence or withdrawal of steroid use has also been significantly associated with pancreatitis in many studies [12]. Hopkins reported that appropriate treatment with corticosteroids improved the survival rate of AP cases associated with SLE. In the Yanget al. series, 16 cases of SLE-related AP were treated with intensive corticosteroid therapy, more or less immunosuppressive therapy, of which 75% had positive outcome [3]. Our two patients showed initial worsening and then a rapid decline in their hemodynamic state with multiorgan failure.

## 5. Conclusion

AP is a rare manifestation of SLE; however, it remains linked to high mortality. This mortality is even more important in case of multiple-organ involvement. The activities of lupus disease and hematological and renal damage worsen the already critical prognosis of lupus-related acute pancreatitis. The early diagnosis of SLE-related AP, suspected in front of any abdominal pain, and appropriate treatment with glucocorticoids are necessary for proper management and prognosis improvement in these patients.
